# Supplementary material for: Abdominal organ segmentation via deep diffeomorphic mesh deformations
Source: Sci Rep. 2023 Oct 25;13:18270. doi: 10.1038/s41598-023-45435-2 (PMC10600339; doi:10.1038/s41598-023-45435-2)
Supplement: Supplementary file 1 — Supplementary Legends. [file 41598_2023_45435_MOESM1_ESM.pdf]

File: UNetFlow Abdominal Organs.mp4

Title: UNetFlow Abdominal Organ Deformation Video

Description: The video shows the deformation from the input template to individual abdominal organs predicted by UNetFlow based on an exemplary scan of the CT test set. Vertices are colored uniquely to allow for the identification of point-wise correspondence between the template and the output shapes.
